# Effects of Behavioral Interventions for Salt Reduction on Blood Pressure and Urinary Sodium Excretion: A Systematic Review and Meta-Analysis of Randomized Controlled Trials

**DOI:** 10.5334/gh.1281

**Published:** 2023-12-22

**Authors:** Ruilong Xun, Yusi Gao, Shiqi Zhen, Tao Mao, Hui Xia, Hong Zhang, Guiju Sun

**Affiliations:** 1 Key Laboratory of Environmental Medicine and Engineering of Ministry of Education, and Department of Nutrition and Food Hygiene, School of Public Health, Southeast University, Nanjing 210009, China; 2 Institute of Health Education, Jiangsu Provincial Center for Disease Control and Prevention, Nanjing 210009, China

**Keywords:** salt, behavioral intervention, blood pressure, urinary sodium excretion, meta-analysis

## Abstract

Hypertension is a prevalent cardiovascular condition, with excessive sodium intake being a significant risk factor. Various studies have investigated measures to reduce salt intake, including integrated lifestyle interventions and health education. However, the effectiveness of behavioral interventions focused solely on salt reduction remains unclear. This systematic review and meta-analysis aimed to investigate the effects of a behavioral intervention based on salt reduction on blood pressure and urinary sodium excretion.

A comprehensive search of the Cochrane Central Register of Controlled Trials, EMBASE, PubMed, and Web of Science was conducted to identify relevant literature. Study and intervention characteristics were extracted for descriptive synthesis, and the quality of the included studies was assessed. A total of 10 studies, comprising 4,667 participants (3,796 adults and 871 children), were included. The interventions involved the provision of salt-restriction spoons or devices, salt-reduction education, self-monitoring devices for urinary sodium, and salt-reduction cooking classes. Meta-analysis results showed that behavioral interventions focused on salt reduction significantly reduced systolic blood pressure (SBP) (–1.17 mmHg; 95% CI, –1.86 to –0.49), diastolic blood pressure (DBP) (–0.58 mmHg; 95% CI, –1.07 to –0.08) and urinary sodium excretion (–21.88 mmol/24 hours; 95% CI, –32.12 to –11.64).

These findings suggest that behavioral change interventions centered on salt reduction can effectively lower salt intake levels and decrease blood pressure levels. However, to enhance effectiveness, behavioral interventions for salt reduction should be combined with other salt-reduction strategies.

## Background

The global burden of cardiovascular diseases (CVDs) has been increasing, with hypertension recognized as one of the most prevalent cardiovascular conditions. In 2019, high systolic blood pressure (SBP) affected 4.06 billion adults worldwide, leading to 10.8 million deaths [1]. Consequently, the prevention and management of hypertension are crucial in addressing this public health concern.

Numerous studies have demonstrated the association between excessive sodium intake and elevated blood pressure [2,3,4]. Excess sodium alters electrolyte balance through its osmotic action [5]. High plasma sodium levels contribute to the entry of large amounts of extracellular fluid, which increases the volume of blood, leading to higher osmolality and blood pressure [5,6]. Moreover, chronic high sodium intake has been linked to organ damage in the heart, kidneys, skin, brain, and bones [7,8,9]. Reducing sodium intake is considered a cost-effective approach to improving global public health [10,11]. Therefore, salt reduction has become a key priority in various strategies aimed at preventing CVDs [12]. The World Health Organization (WHO) recommends a daily salt intake of <5 g per person. Ninety-six countries have implemented national salt-reduction strategies to promote whole population salt reduction in 2019 [13], encompassing interventions in various settings, food reformulation, consumer education, front-of-pack labeling, and salt taxation [13,14].

Numerous original studies have examined the effects of population-based salt-reduction interventions, and relevant systematic reviews and meta-analyses have provided evidence suggesting that reducing sodium intake leads to a decrease in blood pressure [15,16]. However, most of these studies have primarily focused on the relationship between dietary interventions for salt reduction (such as limiting dietary salt intake, providing low-salt foods, salt substitution, and following a healthy dietary pattern) and health outcomes, including intervention effectiveness. As a result, there is a dearth of reviews specifically examining behavioral interventions, with the few available reviews predominantly utilizing urinary sodium excretion as an indicator for evaluating the interventions’ effects. Moreover, many of the interventions discussed in these reviews heavily emphasize comprehensive lifestyle interventions (a combination of interventions such as exercise, healthy diet, and changing bad habits) while neglecting the specific impacts of behavioral interventions targeting salt reduction [17,18,19,20]. Consequently, there is a need to investigate the effects of behavioral approaches to salt reduction, with this study aiming to address this research gap.

This review aimed to summarize interventions that target salt reduction and conduct a meta-analysis to investigate the effects of behavioral interventions, with a specific emphasis on salt reduction, on blood pressure and urinary sodium excretion.

## Methods

### Literature search

The systematic review and meta-analysis were reported according to the Preferred Reporting Items for Systematic Review and Meta-analysis (PRISMA) guidelines (**Supplementary Materials: Appendix A**) [21].

We developed a search strategy to search electronic databases for randomized controlled trial: Cochrane Central Register of Controlled Trials, EMBASE, PubMed, and Web of Science from the start date of the databases to April 2022. Search terms were as follows: salt OR sodium OR “sodium chloride”; restrict* OR reduce* OR minim* OR limit* OR curb* OR intervention OR low* OR free; “blood pressure” OR “urine sodium”; trial (**Supplementary Materials: Appendix B**). Additionally, we reviewed reference lists of relevant original and review articles to search for more trials. There were no language restrictions in the included studies. The search strategy is available in the supplementary materials.

### Study selection

Two authors independently screened abstracts and full texts to evaluate studies of behavior change interventions focused on reducing salt intake. Any disagreements in selections were discussed until a consensus was reached.

The inclusion criteria was developed according to the PICOS approach: (1) population: adult or children (including people in health, hypertension, or prehypertension); (2) intervention: reducing salt intake and interventions aimed at raising awareness and promoting behavioral engagement directly related to salt; (3) comparison: routine care, routine education, or no treatment; (4) outcome: systolic blood pressure (SBP), diastolic blood pressure (DBP), or urinary sodium (estimated by 24-hour urine collection); and (5) setting/design: randomized controlled trials with parallel or crossover design.

The following studies were excluded: cytological studies, animal trials, review articles, repeated literature, or mechanism studies; research not conducted in humans; inappropriate control group; nonbehavioral salt-reduction measures (comprehensive salt-reduction measures, dietary interventions, etc.); personalized intervention; the analytical method was not provided; and lack of access to full texts.

### Data extraction

From each study, we obtained information about author’s name, year of publication, country, characteristics of participants (mean age/size), intervention description, intervention duration, blood pressure at baseline, and changes from baseline (mean/standard deviation). Data on urinary sodium were also collected if they were reported in the included studies. Disagreements between reviewers regarding information abstraction were resolved through discussion. All variables were extracted by two authors independently.

### Quality evaluation

Quality evaluation was assessed independently by two reviewers based on the seven domains defined by the Cochrane Collaboration’s tool for assessing risk of bias, including random sequence generation, allocation concealment, blinding of participants and personnel, blinding of outcome assessment, incomplete outcome data, selective reporting, and other bias.

### Statistical analysis

As for meta-analysis, the mean difference and standard deviation (SD) values of each study were extracted to calculate the continuous variables. The unit of SBP and DBP was shown by ‘mmHg’. The unit of 24-hour urinary sodium was shown by ‘mmol/24 hours’.

All analyses were conducted by using the Stata15.1 based on all included studies. Weighted mean difference (WMD) and 95% confidence interval (95% CI) were used in the calculation. The statistical heterogeneity across the included studies was assessed by using the I^2^ statistic. If the homogeneity of the included studies is <50%, the fixed-effect model is adopted; otherwise, the random effect model is adopted. The low, medium, and high levels of heterogeneity were represented by 25%, 50%, and 75% of I^2^ statistic. Furthermore, sensitivity analysis and subgroup analysis were used to verify the stability of the pooled effects and to explore the main sources of heterogeneity, respectively. The Egger’s test was conducted to detect potential publication bias with significance when *p* < 0.05.

### Certainty assessment

The certainty of evidence was assessed by using the GRADE profiler 3.6 according to Grading of Recommendations Assessment, Development, and Evaluations (GRADE) [22].

## Results

### Characteristics of included studies

**Figure 1:**
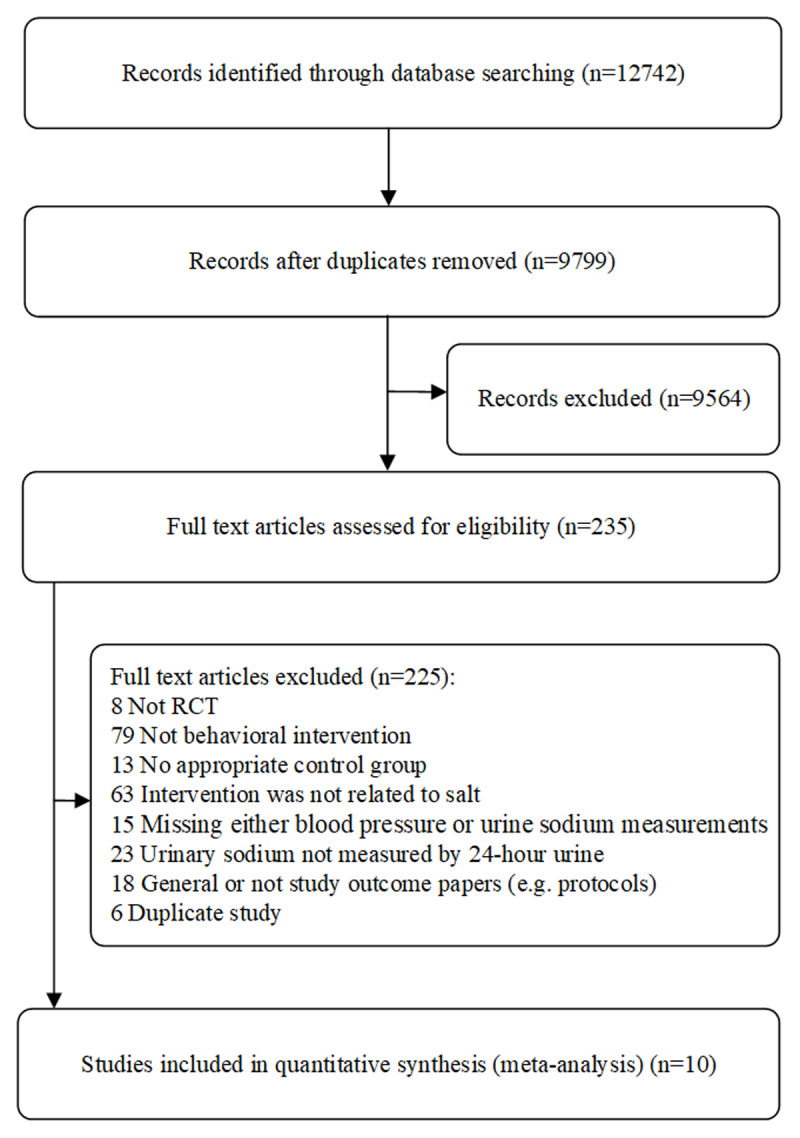
Flow chart of included studies.

The search identified 12,742 records. After screening titles and abstracts, we selected 235 publications for full-text review, of which 225 were excluded for the reasons summarized in Figure 1. A total of 10 studies conformed to the retrieval strategy [23,24,25,26,27,28,29,30,31,32]. No crossover studies were eligible for inclusion. The basic characteristics of subjects and trials were provided in Table 1.

**Table 1:** Characteristics of included studies and results.


LITERATURE	COUNTRY	MEAN AGE (YEARS)	PARTICIPANTS	INTERVENTION DESCRIPTION	INTERVENTION DURATION	BASELINE SBP (MMHG)	CHANGES FROM BASELINE		

							SBP (MMHG)	DBP (MMHG)	URINARY SODIUM (MMOL/24 HOURS)

Kumanyika 2005	USA	43.7 ± 6.2	1159 participants (956 white and 203 black adults) with overweight (577 control, 582 intervention)	C: usual care;I: received comprehensive education and counseling about how to reduce sodium intake	36 months	127.5 ± 6.5	C: 0.62 ± 8.61;I: –0.58 ± 9.06	C: –2.85 ± 6.63;I: –3.04 ± 6.51	C: –10.50 ± 88.5;I: –50.90 ± 86.30

Chen 2013	China	53.5 ± 12.9	403 participants between 40 and 49 years of age (177 control, 226 intervention)	C: no measures;I: received salt restriction spoon+health education	6 months	NA	NA	NA	C: –33.65 ± 98.53;I: –34.84 ± 100.07

He 2015	China	10.1 ± 0.5	279 healthy primary school students in grade 5 (138 control, 141 intervention)	C: no measures;I: received salt reduction education	3.5 months	106.2 ± 11.8^a^	C: 4.40 ± 11.75;I: 3.80 ± 11.87	C: 3.40 ± 12.92;I: 2.40 ± 13.06	C: 20.50 ± 61.09;I: –12.00 ± 60.56

		43.8 ± 12.2	553 adult family members of students (275 control, 278 intervention)	C: no measures;I: received the salt reduction education by children	3.5 months	125.6 ± 25.0	C: 5.00 ± 24.87;I: 3.40 ± 25.01	C: 3.30 ± 17.47;I: 2.70 ± 16.67	C: 13.20 ± 111.95;I: –36.60 ± 111.71

Irwan 2016	Indonesia	66.5 ± 6.1	45 participants with hypertension/prehypertension (17 control, 13 SRT, 15 SREM)	C: no measures;SRT: received salt reduction educational trainingSREM: received salt reduction educational training and a maintenance meeting	1.5 months	145.8 ± 23.3	C: –6.30 ± 20.26;SRT: –9.40 ± 16.52;SREM: –7.70 ± 27.14	C: 0.50 ± 10.41;SRT: –2.80 ± 11.14; SREM: –4.40 ± 13.58	NA

Takada 2016	Japan	63.8 ± 10.84	68 participants (35 housewives and 33 family members) (32 control, 36 intervention)	C: no measures;I: received cooking classes focusing on salt reduction	2 months	133.8 ± 18.6^a^	C: 7.88 ± 11.01;I: 5.05 ± 12.75	C: 3.81 ± 10.47;I: 2.91 ± 7.03	NA

Iwahori 2018	Japan	54.5 ± 8.1	92 participants between 40 and 49 years of age (46 control, 46 intervention)	C: brief dietary education+receive a leaflet as usual care;I: self-monitoring urinary sodium-to-potassium ratio device+brief dietary education+a leaflet as usual care	1 month	125.9 ± 2.2^a^	C: –2.20 ± 8.50;I: –3.4 ± 9.70	C: –1.00 ± 4.20;I: –1.70 ± 6.00	C: –8.70 ± 82.2;I: –18.50 ± 63.60

Yasutake 2018	Japan	58.1 ± 17.4	78 local resident volunteers (36 control, 42 intervention)	C: without any measures;I: used the self-monitoring urinary salt-excretion device	4 weeks	124.3 ± 16.8	C: 2.00 ± 17.96;I: –0.4 ± 16.99	C: –0.40 ± 10.19;I: –0.40 ± 10.47	C: –2.30 ± 65.52;I: –21.17 ± 66.83

Yasutake 2019	Japan	20.8 ± 0.9	100 healthy female college students (49 control, 51 intervention)	C: without any measures;I: used the self-monitoring urinary salt-excretion device	4 weeks	101.4 ± 7.5	C: –2.60 ± 7.11;I: –0.30 ± 7.91	C: –2.40 ± 6.65;I: –1.00 ± 6.76	C: 2.65 ± 52.00;I: –13.70 ± 44.76

Silva 2021	Portugal	48 ± 11	114 workers from a public university (57 control, 57 intervention)	C: no measures;I: used the Salt Control H equipment at home to control salt quantity for cooking all meals	8 weeks	127.0 ± 18.1	C: –3.70 ± 13.94;I: –3.20 ± 14.32	C: –4.80 ± 7.16;I: –4.80 ± 7.16	C: 2.17 ± 61.86;I: –14.61 ± 63.41

He 2022	China	8.6 ± 0.4	592 healthy primary school students in grade 3 (295 control, 297 intervention)	C: no measures;I: received salt reduction education and monitoring via the app-based platform	12 months	93.9 ± 9.4^a^	C: 2.80 ± 9.30;I: 1.50 **±9.80**	C: 1.50 ±7.83;I: 0.20 **±8.17**	C: 9.00 ± 37.16;I: 2.80 **± 36.2**

		45.8 ± 12.9	1184 adult family members of students (590 control, 594 intervention)	C: no measures;I: received salt reduction education and monitoring via the app-based platform	12 months	118.7 ± 17.1	C: –0.40 ± 17.16;I: –3.00 ± 17.08	C: –0.90 ± 10.62;I: –2.20 ± 10.27	C: –4.00 ± 64.19;I: –20.10 ± 57.49


*SBP, systolic blood pressure; DBP, diastolic blood pressure; I, intervention group; C, control group; NA, not applicable; a: reporting using standard validated devices measuring blood pressure.

Randomized controlled trials were published from 2005 to 2022. The studies were conducted in the USA, China, Indonesia, Japan, and Portugal. A total of 4,667 participants were represented in the 10 studies, including 3,796 adults and 871 children; 2,375 females and 2,318 males were involved in these studies, including 3,394 Asians, 1,159 North Americans, and 114 Europeans. These studies measured blood pressure or 24-hour urinary sodium between the intervention group and control groups. All data on urinary sodium were measured from collected 24-hour urine. The length of intervention ranged from four weeks to 36 months.

In terms of intervention strategies, two studies supplied participants with salt-restriction spoons or devices, four studies provided participants with salt-reduction education, three studies supplied participants with self-monitoring devices for urinary sodium, and one study provided cooking classes that focused on salt reduction.

### Risk of bias of included studies

The results of the literature quality evaluation are shown in Table 2. All of the included studies were randomized controlled trials, with no selective reporting and no other bias. Allocation concealment was mentioned in only three articles, and two articles were single-blind (i.e., the subject is blind).

**Table 2:** Cochrane risk of bias assessment tool.


REFERENCE	RISK OF BIAS ASSESSMENT						

	RANDOM SEQUENCE GENERATION	ALLOCATION CONCEALMENT	BLINDING OF PARTICIPANTS AND PERSONNEL	BLINDING OF OUTCOME ASSESSMENT	INCOMPLETE OUTCOME DATA	SELECTIVE REPORTING	OTHER BIAS

Kumanyika 2005	Unclear	Low	High	Low	Low	Low	Low

Chen 2013	Unclear	Low	Low	Low	Low	Low	Low

He 2015	Low	Low	Low	Low	Low	Low	Low

Irwan 2016	Unclear	Low	High	Low	Low	Low	Low

Takada 2016	Unclear	Low	High	Low	Low	Low	Low

Iwahori 2018	Low	Low	High	Low	Low	Low	Low

Yasutake 2018	Unclear	Low	High	Low	Low	Low	Low

Yasutake 2019	Unclear	Low	High	Low	Low	Low	Low

Silva 2021	Low	Low	High	Low	Low	Low	Low

He 2022	Low	Low	Low	Low	Low	Low	Low


The most frequent biases were in the aspects of participant blindness. In some behavioral interventions, such as self-monitoring devices for urinary sodium, participant blindness was not easily achieved. In addition, several studies did not report whether random sequence generation was used.

### Meta-analysis of effects on blood pressure

**Figure 2:**
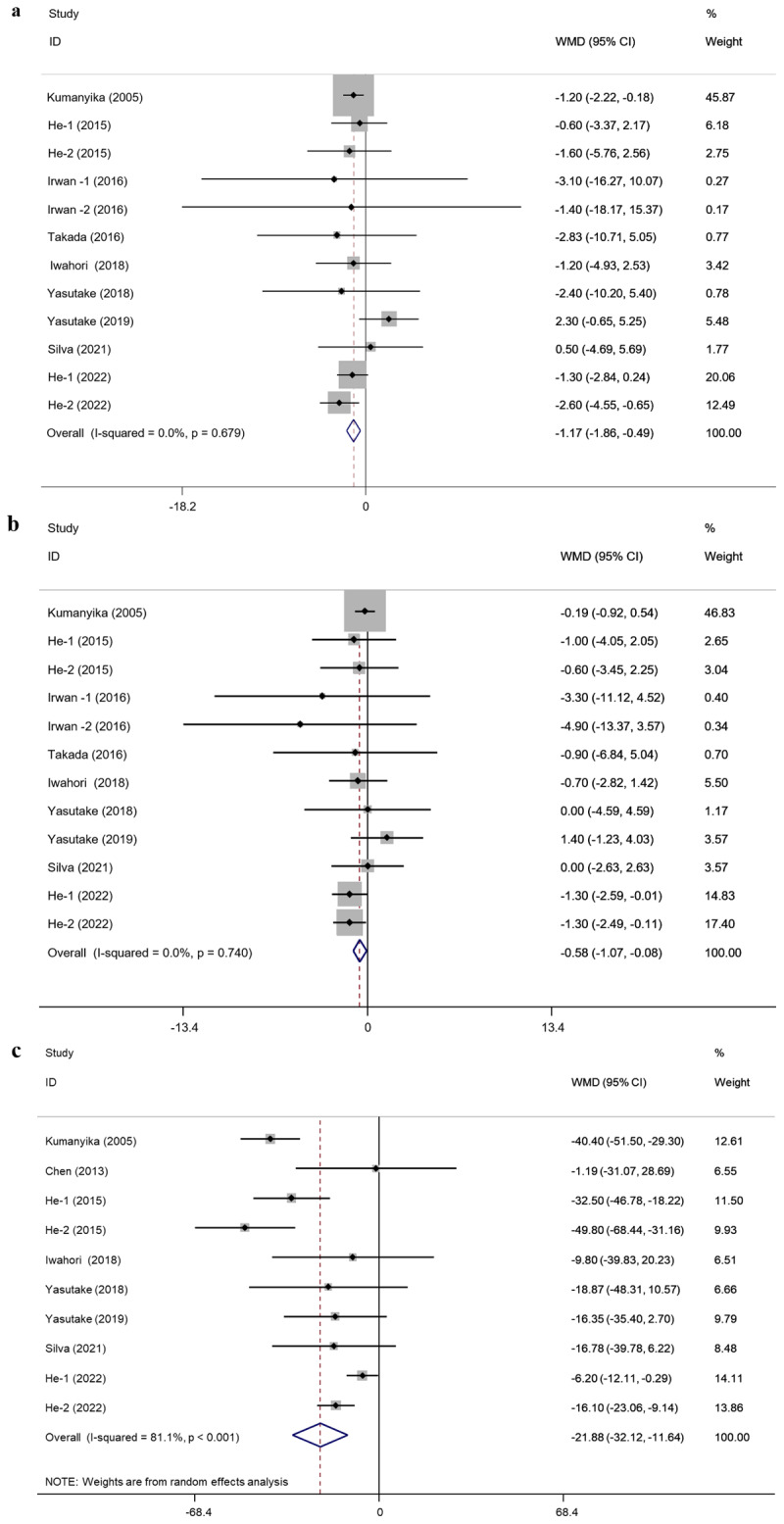
Mean net change in SBP **(a)**, DBP **(b)**, and urinary sodium **(c)** levels and corresponding 95% CI by trial and pooled. SBP, systolic blood pressure; DBP, diastolic blood pressure.

A total of 13 sets of SBP and DBP outcomes were reported in 10 included studies (Figure 2a and 2b). The fixed-effect meta-analysis results indicated that participants with salt-reduction behavioral intervention had lower SBP levels than the control group (WMD = –1.17, 95% CI = [–1.86, –0.49], *p* = 0.001) (Figure 2a). The 13 sets of results showed no statistically significant heterogeneity (I^2^ = 0.0%, *p* = 0.679) (Figure 2a).

In addition, the results showed that individuals with the behavioral intervention of salt reduction had lower DBP levels than the control group (WMD = –0.58, 95% CI = [–1. 07, –0.08], *p* = 0.023) (Figure 2b). The results showed no statistically significant heterogeneity (I^2^ = 0.0%, *p* = 0.740) (Figure 2b).

### Meta-analysis of effects on urinary sodium

Eight included studies reported data on 10 sets of 24-hour urine sodium. The random effect model analysis was applied. The results indicated that the urinary sodium levels of the intervention group were lower than that of the control group (WMD = –21.88, 95% CI = [–32.12, –11.64], *p* < 0.001) (Figure 2c). The results showed a statistically significant amount of heterogeneity (I^2^ = 81.1%, *p* < 0.001) (Figure 2c).

### Subgroup analysis

**Figure 3:**
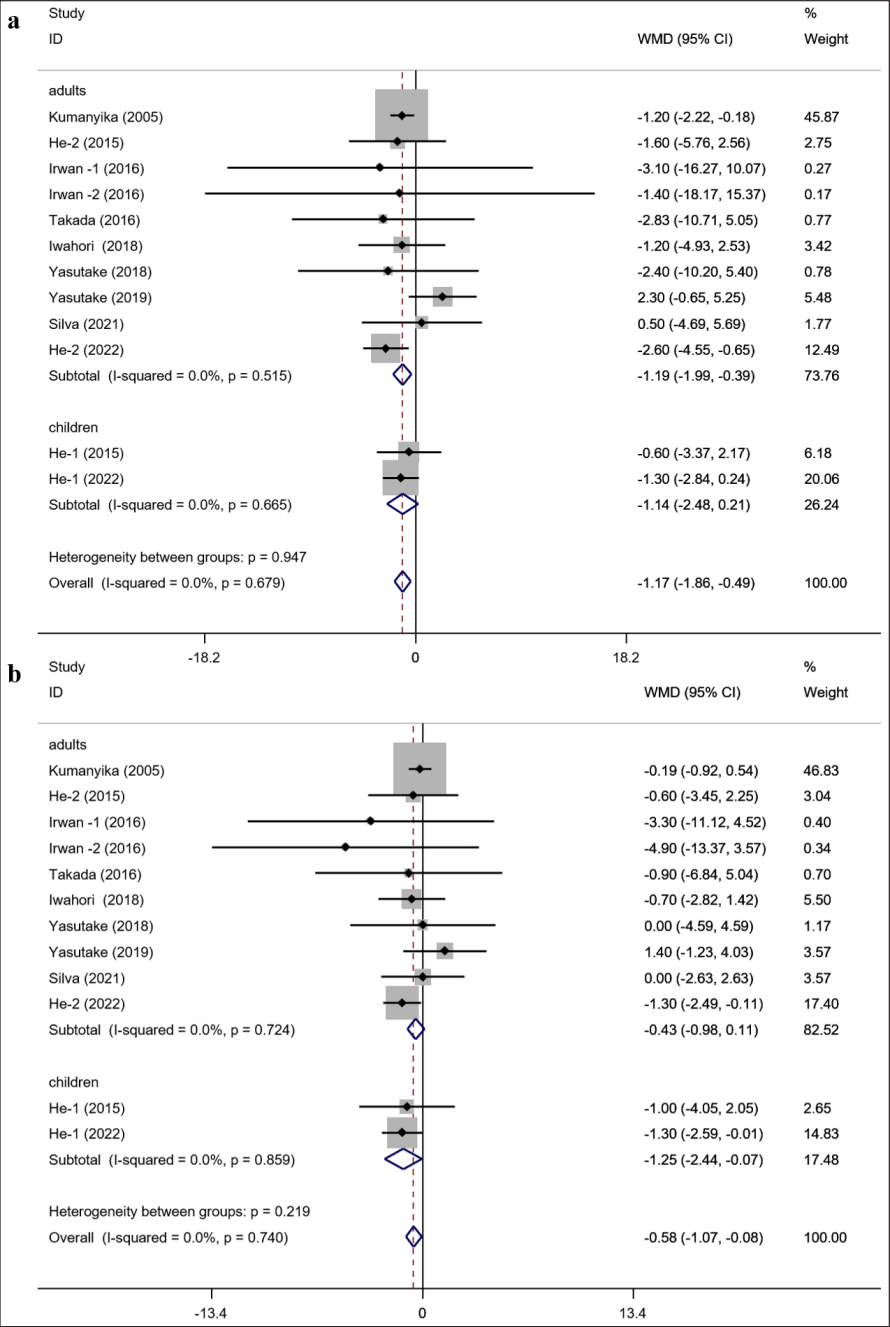
The results of subgroup analysis of SBP and DBP: **(a)** subgroup analysis of SBP by adult or child subgroup; **(b)** subgroup analysis of DBP by adult or child subgroup. SBP, systolic blood pressure; DBP, diastolic blood pressure.

Subgroup analyses of SBP, DBP, and urinary sodium levels are shown in the **Supplementary Materials: Table S1–2**. Since both children and adults were collected in the included studies, subgroup analyses of SBP and DBP were performed according to it. The results showed no detectable heterogeneity (Figure 3a and 3b).

**Figure 4:**
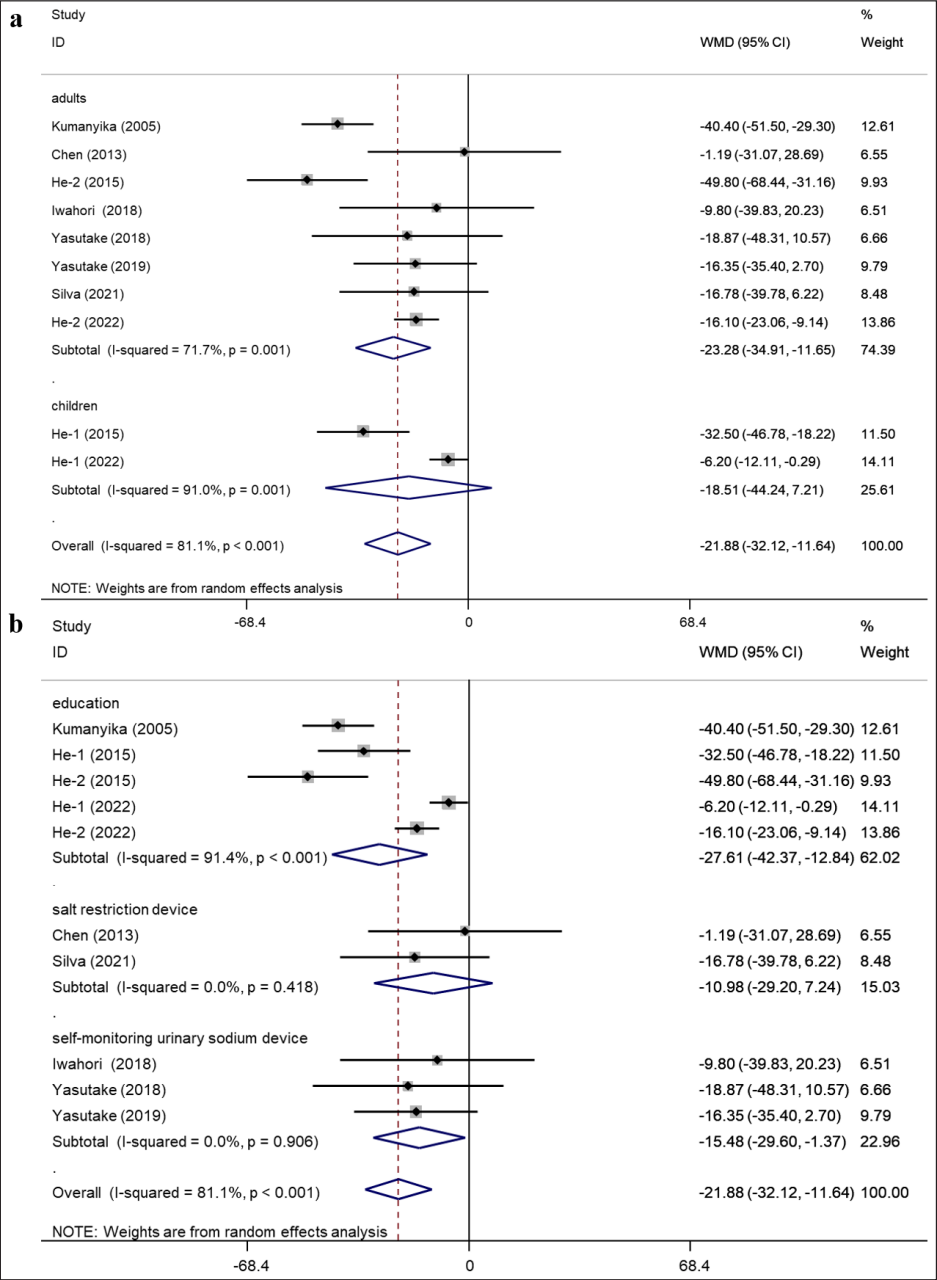
The results of subgroup analysis of urinary sodium: **(a)** subgroup analysis of urinary sodium by adult or child subgroup; **(b)** subgroup analysis of urinary sodium by invention type subgroup.

In the analysis of urinary sodium, subgroup analysis was conducted according to five factors: average age, adults or children, intervention duration, intervention type, and baseline SBP. The subgroup analysis of urinary sodium suggested that the average age, adults or children, intervention duration, intervention type, and baseline SBP may not be sources of high heterogeneity (**Supplementary Materials: Table S3**). Results of subgroup analysis of urinary sodium grouped by adult (age ≥ 18) or child (age < 18) and intervention type are additionally presented (Figure 4a and 4b).

Subgroup analysis showed that behavioral interventions significantly reduced SBP and urinary sodium levels in adults and DBP and urinary sodium levels in children. Salt-reduction education interventions significantly reduced SBP, DBP, and urinary sodium levels. Intervention with a self-monitoring urinary sodium device significantly reduced urinary sodium levels.

### Sensitivity analysis

Sensitivity analysis was carried out by eliminating one included study at a time in turn. Sensitivity analysis results of SBP, DBP, and urinary sodium levels did not have substantial changes, indicating that the results of the meta-analysis were relatively reliable (**Supplementary Materials: Figure S2**).

### Publication bias

Egger’s regression was used to examine publication bias with significance when the *p* value is <0.05. There was no evidence of possible publication bias for SBP (*p* = 0.819), DBP (*p* = 0.495), and urinary sodium (*p* = 0.298).

### Certainty of evidence

The certainty of evidence was assessed as moderate in the SBP and DBP outcomes and low in the urinary sodium outcome (Table 3).

**Table 3:** Certainty of evidence of systolic blood pressure, diastolic blood pressure, and urinary sodium assessment.


QUALITY ASSESSMENT							NO. OF PATIENTS		EFFECT		QUALITY	IMPORTANCE
		
NO. OF STUDIES	DESIGN	RISK OF BIAS	INCONSISTENCY	INDIRECTNESS	IMPRECISION	OTHER CONSIDERATIONS	BEHAVIORAL INTERVENTION BASED ON SALT REDUCTION	CONTROL	RELATIVE(95% CI)	ABSOLUTE		

**Systolic blood pressure (follow-up 1–36 months; better indicated by lower values)**												

12	Randomized trials	Serious^1^	No serious indirectness	No serious indirectness	No serious imprecision	None	2173	2154	–	WMD 1.17 lower (0.49–1.86 lower)	⊕⊕⊕**O**Moderate	Critical

**Diastolic blood pressure (follow-up 1–36 months; better indicated by lower values)**												

12	Randomized trials	Serious^1^	No serious indirectness	No serious indirectness	No serious imprecision	None	2173	2154	–	WMD 0.58 lower (0.08–1.07 lower)	⊕⊕⊕**O**Moderate	Critical

**Urinary sodium (follow-up 1–36 months; better indicated by lower values)**												

10	Randomized trials	Serious^1^	Serious^2^	No serious indirectness	No serious imprecision	None	2314	2222	–	WMD 21.88 lower (11.64–32.13 lower)	⊕⊕⊕**O**Low	Critical


^1^Allocation concealment was mentioned in only three articles, and two articles were single-blind. ^2^High heterogeneity.

## Discussion

The present study highlights the importance of salt-reduction behavioral interventions for adherence to lifestyle change. Our meta-analysis suggests that such interventions can effectively decrease blood pressure and urinary sodium excretion.

The moderate-quality estimated pooled effect sizes for the effects on SBP and DBP were significantly –1.17 mmHg and –0.58 mmHg, respectively. The intervention has shown a better effect on lowering blood pressure in adults than in children. The intervention can lower blood pressure, although the effect was not large. The majority of our included studies had participants with normal blood pressure, and only one study (45 participants included) had mean blood pressure above 140 mmHg at baseline, which may explain the small blood pressure–lowering effect.

Moreover, the low-quality estimated pooled effect size for the effect on urinary sodium was significantly –21.88 mmol/24 hours. Translated to sodium intake, it indicated a 0.5 g/day reduction in sodium intake relative to an estimated reduction in salt intake of 1.3 g/day, equating to an estimated reduction in salt intake of 1.3 g/day. It has been shown that a 2 g/day reduction in salt intake can reduce the incidence of hypertension by 35% in individuals with normal blood pressure [33]. Therefore, salt-reduction behavioral interventions provide significant implications for public health. However, for participants with hypertension or prehypertension, much greater salt reduction may be necessary to decrease blood pressure and relieve the disease. Subgroup analyses indicated that salt-reduction education and urinary sodium self-monitoring were effective and better with educational interventions.

In this study, we used data from 24-hour urinary sodium to assess daily sodium intake, as this is considered the gold standard method [34] with reliable outcomes. Data collected via point urine or overnight eight-hour urine was excluded, as the estimated sodium intake is not as accurate, which may lead to inconsistent bias [35]. In our study, behavioral interventions decreased urinary sodium levels to a greater extent than reducing blood pressure. Possibly because this study mainly included healthy people, a decrease in sodium intake had a relatively small effect on blood pressure reduction in the nonhypertensive population, similar to other studies [15,36]. It is also possible that the intervention duration of studies was generally insufficient, mostly no longer than 12 months. More significant changes in blood pressure may take a longer period of intervention duration.

The results of salt-reduction education were significant, with an impact on participants’ awareness of salt reduction. Nevertheless, continuous monitoring of educational interventions is required. Salt-reduced cooking courses for housewives were targeted and profitable [27], providing ideas for community hypertension prevention. Salt-reduction education in schools combined with families deserved to be promoted and was effective for both children and families [23]. This may be due to parents making a conscious effort to reduce sodium intake in their family life, such as using less salt in cooking or purchasing low-salt packaged foods [37]. Salt-reduction education can also be combined with environmental support to enhance the effectiveness of the intervention by providing a supportive food environment for salt-reduction education [38], such as the promotion of low-salt diets in canteens and salt labels for packaged foods [39].

The urinary sodium self-monitoring intervention, although beneficial in developing low-salt habits, is difficult to implement in a large population due to the instrument’s cost and inconvenience. Cheaper and more convenient instruments may make it an effective way to adopt low-salt habits in people with high blood pressure and high risk. The combination of this intervention with salt education is also required to raise awareness of salt reduction, since the consumption of salty foods may increase when people reduce their salt consumption [25,31].

Generally comprehensive intervention studies—in other words, interventions that did not focus on salt reduction—were excluded. These interventions did not highlight the importance of salt reduction, nor could they estimate the effect of salt-reduction interventions alone. Studies such as salt substitution, low-sodium foods, and the Dietary Approaches to Stop Hypertension (DASH) diet were also excluded because of the dietary intervention rather than salt-reduction interventions designed to promote behavior. Salt substitutes are mainly salt, with potassium chloride replacing sodium chloride. Low sodium and high potassium intake could help lower blood pressure and may reduce the incidence of cardiovascular disease [40]. The DASH diet contains whole grain vegetables, fruits, lean meats, and fat-free dairy products, in addition to a few micronutrients. The DASH diet is nutritious, low in sodium, and seems to exert natriuretic action that can help lower blood pressure [20,41].

Obviously, these dietary interventions can decrease salt intake and tend to be more effective. But it takes greater resources for the government. Behavioral interventions such as education and cooking training are low cost, widely accessible, and highly feasible, though less effective. Additionally, studies providing personalized salt-reduction education or counseling were ruled out, as this would be difficult to replicate directly in a large population.

We compared systematic reviews and meta-analyses related to salt-reduction interventions, most of which were published after 2013, with studies including dietary sodium reduction, salt substitution, salt-reduction behaviors, and all salt-reduction interventions. These reviews generally only included adults, while our study included both adults and children to explore the effects of salt-reduction behavioral interventions on all age groups. However, only two met the requirements, namely HE 2015 [24] and HE 2022 [32]. The results showed that salt-reduction education decreased SBP and reduced sodium intake in children. Implementing salt-reduction behavioral measures from childhood may reduce hypertension in adulthood [42,43], and knowledge and behavioral habits acquired during childhood are likely to persist into adulthood [44]. More cohort studies on behavioral interventions for salt reduction in children are needed to provide more evidence.

High heterogeneity was detected when analyzing the effect on urinary sodium excretion. Subgroup analyses were conducted separately by average age, adults or children, intervention duration, intervention type, and baseline SBP. However, none of these was a source of heterogeneity. The effect of salt-reduction behavior may also be influenced by geographical reasons, cultural background, dietary patterns, and others. It is worth noting that there appears to be an interaction between intervention duration and intervention type, with educational interventions preferring longer intervention durations.

This meta-analysis still has limitations that need to be noted. The number of studies we included was small, and the study only included behavior change interventions that focused on salt reduction, excluding those that only included but did not highlight it; for example, healthy dietary education to prevent hypertension was excluded. Our findings lacked high-quality results to support the conclusions, and large heterogeneities emerged in the analysis beyond explanation. In addition, this meta-analysis was not registered and did not involve any clinical outcomes, making it difficult to analyze whether salt-reduction interventions have long-term clinical significance in hypertensive patients, as most of the studies included were in nonhypertensive populations.

Reducing salt intake in people with hypertension requires more coercive means to reduce sodium intake, whereas nonhypertensive people may not have this awareness, and salt reduction behavior is autonomously selective. The study included mostly nonhypertensive participants, making it generalizable to healthy populations with a high significance for reducing the risk of hypertension.

The results of this study imply the effectiveness of behavioral interventions that focus on salt reduction. Behavioral interventions are one cost-effective measure to promote people’s health [45]. However, it is worth noting that to meet the WHO target of 5 g salt intake, countries must implement comprehensive salt-reduction measures, such as limiting the salt content of processed foods and the use of salt in the catering industry [46].

## Conclusions

In summary, this meta-analysis shows that behavioral interventions based on salt reduction can decrease SBP, DBP levels, and 24-hour urinary sodium levels. Although the clinical significance of these reductions may be limited, they can still have important public health implications by helping to reduce people’s daily salt intake. However, it should be noted that these interventions alone may not be sufficient for effective prevention of hypertension and may need to be combined with other salt-reduction measures. Larger population studies with longer durations are needed to further explore the patterns and effects of behavioral interventions for salt reduction.

## Data Accessibility Statement

The datasets used or analyzed during the current study are available from the corresponding author on reasonable request.

## Additional Files

The additional files for this article can be found as follows:

10.5334/gh.1281.s1 Supplementary Materials. Appendix A: PRISMA Checklist.

10.5334/gh.1281.s2 Supplementary Materials. Appendix B: Specific Search Methods.

10.5334/gh.1281.s3 Supplementary Materials. Appendix C: Supplementary Tables and Figures.

## Author Information

Correspondence to Guiju Sun and Hong Zhang.
