# Supplementary material for: Effects of Behavioral Interventions for Salt Reduction on Blood Pressure and Urinary Sodium Excretion: A Systematic Review and Meta-Analysis of Randomized Controlled Trials
Source: Glob Heart. 2023 Dec 22;18(1):65. doi: 10.5334/gh.1281 (PMC10742105; doi:10.5334/gh.1281)
Supplement: Supplementary Materials. — Appendix B: Specific Search Methods. [file gh-18-1-1281-s2.pdf]

Appendix B: specific Search methods

Cochrane library (6042 records):

1 salt OR sodium OR "sodium chloride" [Title abstract keywords]

2 restrict* or reduc* or minim* or limit* or curb* or intervention or low* or free [Title abstract keywords]

3 "blood pressure" OR "urine sodium" [All text]

4 #1 AND #2 AND #3

PubMed (2579 records):

((restrict*[Title/Abstract] OR reduc*[Title/Abstract] OR minim*[Title/Abstract] OR limit*[Title/Abstract] OR curb*[Title/Abstract] OR intervention[Title/Abstract] OR low[Title/Abstract] OR free[Title/Abstract]) AND (salt[Title/Abstract] OR sodium[Title/Abstract] OR "sodium chloride"[Title/Abstract])) AND ('blood pressure' OR 'urinary sodium')

EMBASE (1132 records):

1 salt：ab, ti

2 sodium: ab, ti

3 ‘sodium chloride’: ab, ti

4 restrict$ OR reduc$ OR minim& OR limit$ OR curb$ OR intervention OR low$ OR free: ab, ti

5 #1 OR #2 OR #3

6 #5 AND #4

7 #6 AND 'randomized controlled trial'/de

8 ‘urinary sodium’: ab, ti

9 ‘blood pressure’: ab, ti

10 #9 OR #8

11 #7 AND #10

WEB OF SCIENCE (2989 records):

1 salt OR sodium OR “sodium chloride” [Topic]

2 restrict* or reduc* or minim* or limit* or curb* or intervention or low* or free [Abstract]

3 “blood pressure” OR “urinary’ sodium” [Abstract]

4 trial [Abstract]

5 #1 AND #2 AND #3 AND #4
