# Supplementary material for: Effects of Behavioral Interventions for Salt Reduction on Blood Pressure and Urinary Sodium Excretion: A Systematic Review and Meta-Analysis of Randomized Controlled Trials
Source: Glob Heart. 2023 Dec 22;18(1):65. doi: 10.5334/gh.1281 (PMC10742105; doi:10.5334/gh.1281)
Supplement: Supplementary Materials. — Appendix C: Supplementary Tables and Figures. [file gh-18-1-1281-s3.pdf]

Appendix C: Supplementary Tables and Figures

Table S1 Subgroup analysis of systolic blood pressure

Subgroups	No. of trials	WMD (95%CI)	I^2 (%)	*p* value
Average age (years)				
≤18	2	-1.14 (-2.48, 0.21)	0.0	0.098
>18 to ≤45	3	-0.87 (-1.80, -0.07)	59.8	0.070
>45	7	-2.08 (-3.63, -0.52)	0.0	0.001
Population				
Children	2	-1.14 (-2.48, 0.21)	0.0	0.098
Adults	10	-1.19 (-1.99, -0.39)	0.0	0.004
Intervention duration (months)				
≤3	7	0.34 (-1.60, 2.27)	0.0	0.734
>3 to ≤6	2	-0.91 (-3.21, 1.40)	0.0	0.440
>6	3	-1.45 (-2.23, -0.67)	0.0	< 0.001
Intervention type				
Education	8	-1.41 (-2.14, -0.68)	0.0	< 0.001
Salt restriction device	1	0.68 (-1.53, 2.90)	—	0.850
Self-monitoring urinary sodium device	3	0.50 (-4.69, 5.69)	27.0	0.545

Baseline SBP				
<120	3	-0.54 (-1.77, 0.68)	55.7	0.385
>120 to ≤140	7	-1.46 (-2.30, -0.63)	0.0	0.001
> 140	2	-2.45 (-12.87, 7.91)	0.0	0.643

Table S2 Subgroup analysis of diastolic blood pressure

Subgroups	No. of trials	WMD (95%CI)	I^2 (%)	*p* value
Average age (years)				
≤18	2	-1.25 (-2.44 -0.07)	0.0	0.038
>18 to ≤45	3	-0.11 (-0.79, 0.57)	0.0	0.757
>45	7	-1.04 (-1.96, -0.11)	0.0	0.023
Population				
Children	2	-1.25 (-2.44 -0.07)	0.0	0.038
Adults	10	-0.43 (-0.98 -0.11)	0.0	0.119
Intervention duration (months)				
≤3	7	-0.16 (-1.44, 1.11)	0.0	0.801
>3 to ≤6	2	-0.79(-2.87, 1.29)	0.0	0.459
>6	3	-0.64 (-1.20, -0.08)	45.5	0.024
Intervention type				
Education	8	-0.68 (-1.22, -0.15)	0.0	0.012
Salt restriction device	1	0.00 (-2.63, 2.63)	—	1.000
Self-monitoring urinary sodium device	3	0.11 (-1.44, 1.66)	0.0	0.888
Baseline SBP				

<120	3	-0.80 (-1.89, 0.28)	39.1	0.145
>120 to ≤140	7	-0.48 (-1.04, 0.08)	0.0	0.091
> 140	2	-4.04 (-9.78, 1.71)	0.0	0.169

Table S3 Subgroup analysis of urinary sodium

Subgroups	No. of trials	WMD (95%CI)	I^2 (%)	*p* value
Average age (years)				
≤18	2	-18.51 (-44.24, 7.21)	91.0	0.158
>18 to ≤45	3	-36.07 (-53.01, -19.13)	70.0	< 0.001
>45	5	-15.36 (-21.57, -9.15)	0.0	< 0.001
Population				
Children	2	-18.51 (-44.24, 7.21)	91.0	0.158
Adults	8	-23.28 (-34.91, -11.65)	71.7	< 0.001
Intervention duration (months)				
≤3	4	-15.84 (-27.87, -3.81)	0.0	< 0.001
>3 to ≤6	3	-30.64 (-52.92, -8.36)	73.1	< 0.001
>6	3	-20.26 (-36.99, -3.53)	93.0	< 0.001
Intervention type				
Education	5	-27.61 (-42.37, -12.84)	91.4	< 0.001
Salt restriction device	2	-10.98 (-29.20, 7.24)	0.0	0.938

Self-monitoring urinary sodium device	3	-15.48 (-29.60, -1.37)	0.0	0.032
Baseline SBP				
<120	3	-17.58 (-35.12, -0.04)	82.6	0.030
>120 to ≤140	6	-26.79 (-40.74, -12.83)	77.3	< 0.001
NA	1	-1.19 (-31.07, 28.69)	—	0.938

* SBP, systolic blood pressure; NA, not available.

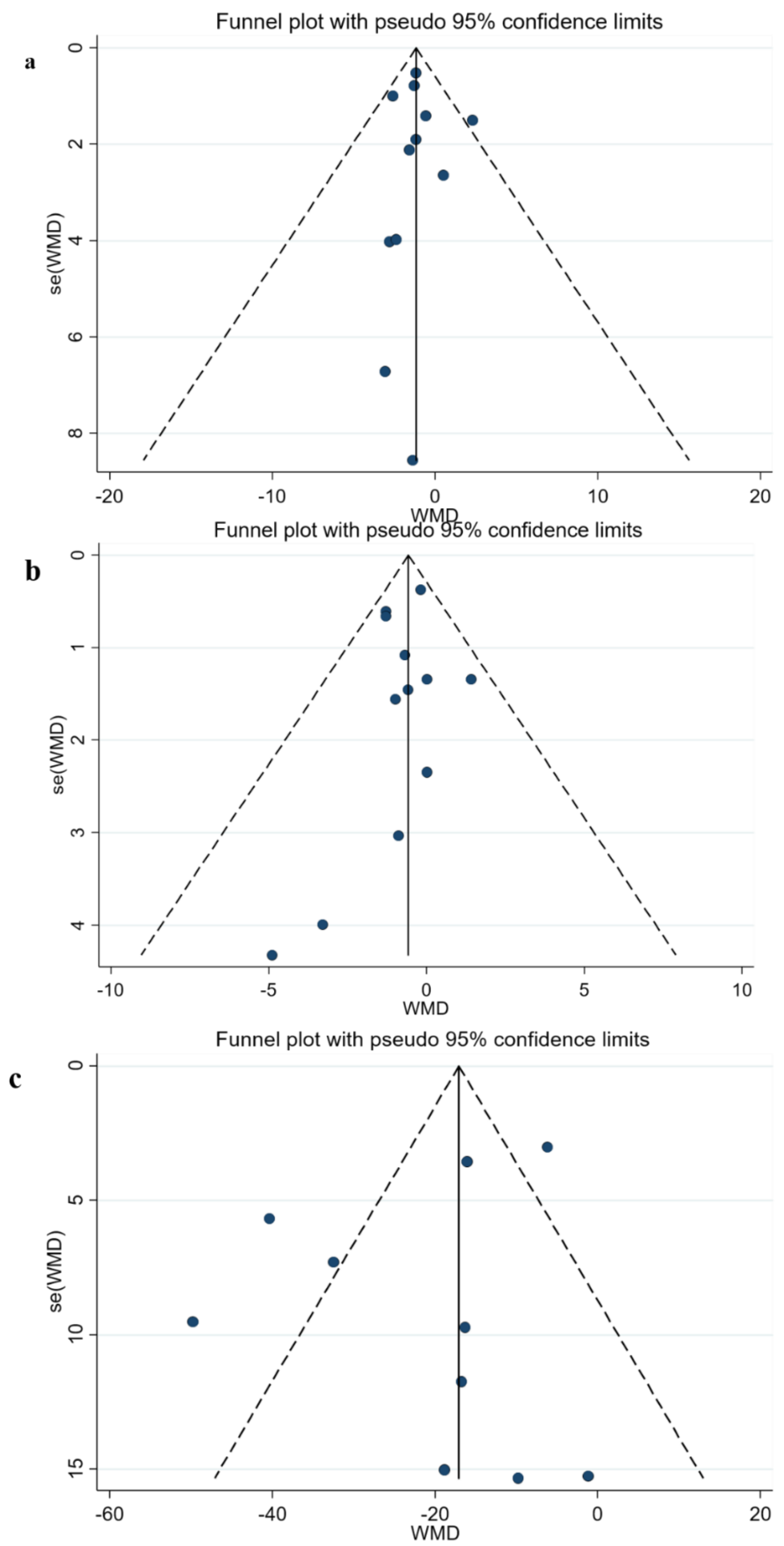

Figure S1 Funnel plots. (a) systolic blood pressure; (b) diastolic blood pressure; (c) urinary sodium.

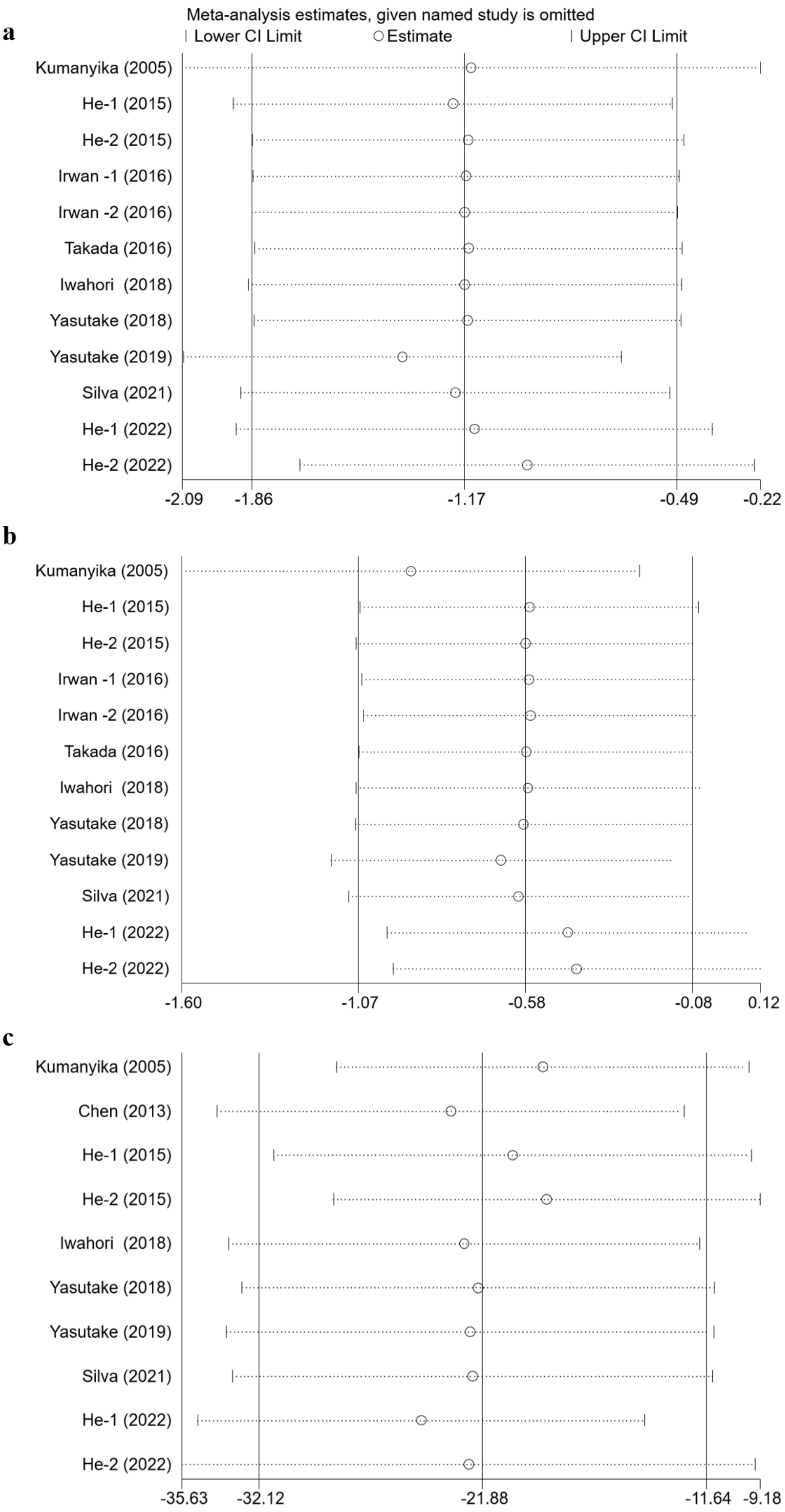
Meta-analysis estimates, given named study is omitted
Lower CI Limit
Estimate
Upper CI Limit
a
Kumanyika (2005)
He-1 (2015)
He-2 (2015)
Irwan -1 (2016)
Irwan -2 (2016)
Takada (2016)
Iwahori (2018)
Yasutake (2018)
Yasutake (2019)
Silva (2021)
He-1 (2022)
He-2 (2022)
-2.09
-1.86
-1.17
-0.49
-0.22
b
Kumanyika (2005)
He-1 (2015)
He-2 (2015)
Irwan -1 (2016)
Irwan -2 (2016)
Takada (2016)
Iwahori (2018)
Yasutake (2018)
Yasutake (2019)
Silva (2021)
He-1 (2022)
He-2 (2022)
-1.60
-1.07
-0.58
-0.08
0.12
c
Kumanyika (2005)
Chen (2013)
He-1 (2015)
He-2 (2015)
Iwahori (2018)
Yasutake (2018)
Yasutake (2019)
Silva (2021)
He-1 (2022)
He-2 (2022)
-35.63
-32.12
-21.88
-11.64
-9.18

Figure S2 Sensitivity analysis. (a) systolic blood pressure; (b) diastolic blood pressure; (c) urinary sodium.
